# Acute pancreatitis adversely impacts the outcome in hospitalized pediatric hematopoietic stem cell transplantation recipients

**DOI:** 10.1038/s41390-025-04753-z

**Published:** 2026-01-16

**Authors:** Aravind Thavamani, Isabella Zaniletti, Matt Hall, Thomas J. Sferra, Jignesh Dalal, Senthilkumar Sankararaman

**Affiliations:** 1 UH Rainbow Babies & Children’s Hospital, Pediatric Gastroenterology, Cleveland, OH USA; 2 Case Western Reserve University School of Medicine, Department of Pediatrics, Cleveland, OH USA; 3 Children’s Hospital Association, Overland Park, KS USA; 4 UH Rainbow Babies & Children’s Hospital, Pediatric Hematology Oncology, Cleveland, OH USA; 5 Cleveland Clinic Children’s Hospital, Pediatric Gastroenterology, Cleveland, OH USA; 6 Department of Pediatrics, Cleveland Clinic Lerner College of Medicine of the CWRU, Cleveland, OH USA

## Abstract

**Background:**

Acute pancreatitis (AP) is one of the serious complications among patients with hematopoietic stem cell transplantation (HSCT). We aimed to evaluate the impact of AP on hospitalization outcomes in pediatric HSCT recipients.

**Methods:**

We retrospectively queried the PHIS database to include all pediatric patients who underwent HSCT between 2005-2024. The study population was divided into those with and without AP, and compared for various demographic and clinical variables. The primary outcome was in-hospital mortality, and secondary outcomes included healthcare resource utilization.

**Results:**

A total of 33,439 HSCT recipients were identified, and the prevalence rate of AP was 1.1%. The AP group had a significantly higher in-hospital mortality rate (22.7% vs. 4.6%), higher median hospitalization costs (USD 698,372 vs. 187,583), and higher median length of stay (138.5 vs. 60 days) (all p < 0.001). Our risk stratification model predicted a 2.7 (95% CI: 2.2 to 3.4) increased risk of AP for a score of >10, with an AUC of 0.85 (95% CI: 0.84 to 0.88).

**Conclusion:**

AP in HSCT prognosticates adverse outcomes with increased in-hospital mortality, prolonged length of stay, and higher hospitalization costs. Clinicians need to be vigilant in preventing and aggressively managing AP in HSCT.

**Category of study:**

Population study

**Impact:**

- Acute pancreatitis (AP) in pediatric hemopoietic stem cell transplant (HSCT) recipients prognosticates adverse outcomes with increased mortality, prolonged length of stay, and higher hospitalization costs.
- The risk stratification model predicted a 2.7 (95% CI: 2.2 to 3.4) times increased risk of AP for a score of >10, with an AUC of 0.85 (95% CI: 0.84 to 0.88).
- Multivariable analysis showed AP was associated with 1.5 (CI:1.2 to 2, p = 0.001) times increased risk of mortality in pediatric population.

## Introduction

Hematopoietic stem cell transplantation (HSCT) is a lifesaving and potentially curable treatment for refractory hematological malignancies, various solid tumors, and several non-malignant disorders such as hematopoietic and inherited metabolic disorders. However, this procedure is not without complications, and acute pancreatitis (AP) is one of the dreaded complications that can develop among patients with HSCT.^1,2^ Pediatric AP has been previously defined as having the presence of at least two of the following three criteria, abdominal pain compatible with pancreatic origin, amylase and/or lipase at least three times upper limits of normal, and imaging findings suggestive/compatible with pancreatic inflammation.^3^

Werlin et al. noted AP in HSCT in the early 1990s (2). Various causes such as medications (including chemotherapeutic, immunosuppressive drugs, and conditioning therapy, graft versus host disease (GVHD), infections (including adenovirus, cytomegalovirus, Epstein-Barr virus, and varicella-zoster virus), gall stones, and radiation therapy have been implicated for AP in patients with HSCT.^1,2,4–17^ AP in HSCT recipients can result in significant morbidity and mortality.^1,2^ In our prior study using 2 national-level databases, we demonstrated that AP-related hospitalizations in HSCT independently increased the risk of in-hospital mortality 3.4 times (95% CI 2.86–4.02, *p* < 0.001) and also increased the length of stay and hospitalization charges.^18^

In a study by Wang et al involving 5284 adults who underwent HSCT, 0.76% developed AP.^4^ In this study, the authors developed a predictive scoring for the development of AP in HSCT. Age (younger adults), GVHD, and choledocholithiasis were incorporated in the predictive scoring for the development of AP.^4^ Similar predictive scoring for the development of AP in pediatric HSCT recipients is not currently existent and we sought to evaluate this risk scoring in a larger sample using a national-level database. Identification of predictive factors and development of similar scoring in pediatrics will help clinicians to be more vigilant in both prevention and aggressive management of AP and may help minimize the adverse outcomes.

Our hypothesis was that AP in HSCT could be adversely associated with increased in-hospital mortality and unfavorable hospitalization outcomes. The primary aim of the study was to evaluate the prevalence of in-hospital mortality in patients with AP and HSCT when compared with HSCT patients without AP. Additionally, we wanted to evaluate the prevalence of other adverse hospital outcomes such as length of hospital stay and hospitalization costs in this cohort. Further, we also wanted to formulate a risk scoring that can be predictive of the development of AP in pediatric HSCT recipients.

## Methodology

In this retrospective study, we analyzed the Pediatric Health Information System (PHIS) database which is an aggregation of inpatient, observation, emergency department, and ambulatory surgery encounter data from more than 49 children’s hospitals across the United States affiliated with the Children’s Hospital Association (CHA, Lenexa, KS).^19^ This database was created for the purposes of external benchmarking and quality improvement; the validity and reliability of the datasets are ensured both by CHA and the partnering hospitals.^19^ The dataset includes patient demographics, admission details, comorbid diagnoses, and procedures based on the *International Classification of Diseases, 9th Revision and 10th Revision, Clinical Modification* (ICD-9/10-CM), and Current Procedural Terminology codes.

Partnering hospitals also submit resource utilization data based on clinical, pharmaceutical, laboratory, and imaging charges. In the PHIS database, data are deidentified at submission and are subjected to reliability and validity checks before inclusion. We analyzed the PHIS database to include all patients who underwent HSCT between 2005 to 2024. The University Hospitals Cleveland Medical Center Institutional Review Board (IRB) reviewed this project and decided it was not a research activity (IRB number: STUDY20210314) and deemed exempt.

## Inclusion and exclusion criteria

All HSCT (all ICD codes are mentioned in Supplemental Table S1) patients under 18 years of age were divided into two group–patients who developed with AP after HSCT (diagnosis of HSCT should precede AP) and this group was referred to as the study group and all HSCT patients without AP diagnosis was termed as the control group. We included both allogeneic- and autologous HSCT pediatric recipients. The occurrence of AP might have occurred either during the same hospitalization for HSCT (occurrence of HSCT should precede before AP) or during subsequent hospitalizations. Both groups were compared for various demographics, detailed clinical characteristics, and comorbid conditions related to both AP and HSCT.^11,18^ Patients with chronic pancreatitis were excluded.

## Outcome measures

The primary outcome was in-hospital mortality. Additionally, we reported the unadjusted association of AP with secondary clinical outcomes, including the length of hospital stay and total hospitalization costs which were used as the surrogates of healthcare resource utilization. Length of hospital stay and costs were calculated across all hospitalizations. PHIS database has charges and costs were estimated from the hospital/year specific cost-to-charge ratio. We evaluated complications such as respiratory failure, use of intubation and mechanical ventilation, hypotension, use of vasopressors, sepsis, multi-organ dysfunction syndrome, acute kidney injury, pulmonary embolism, and deep vein thrombosis between the two cohorts.^11,18^ Further, we evaluated central venous catheter placement, use of parenteral nutrition, complications, and surgeries/procedures associated with AP or HSCT.

## Statistical methods

Categorical variables were summarized as count and percentage, while continuous variables were presented as median and interquartile range (IQR). We compared AP vs non-AP groups in unadjusted analysis using the Chi-Square test and Wilcoxon rank-sum test as appropriate. We calculated the risk of in-hospital mortality for AP using aa multivariable cause-specific Cox proportional hazard model and visually represented the probability of survival over time with adjusted Kaplan-Meier curve. To develop the risk calculator, we modeled the probability of AP using a multivariable generalized linear mixed model (GLIMMIX) for binary distribution and hospitals as clusters. Clinically relevant and statistically significant adjustors for all models included age, race, hypertriglyceridemia, hemophagocytic lymphohistiocytosis (HLH), choledocholithiasis, myelodysplastic, and GVHD.

To develop the risk score, we assigned each factor in multivariable analysis weighted points proportional to the regression coefficient value.^20^ Once the total score was calculated for each individual in the cohort by adding together the points corresponding to their risk factors, cut-off point analysis was conducted to find the value that optimized the receiving operating curve (ROC) based on the distance between such point and the point (0,1). Such value represents the ideal classification that maximizes sensitivity and specificity. We validated the classification using it as exposure in the model for the outcome, reporting the area under the ROC with its 95% confidence interval.

All analyses were conducted in SAS Enterprise Guide v8.3 (SAS, Cary, NC) and statistical significance was set at *p* < 0.05. We adhered to the Strengthening The Reporting of OBservational studies in Epidemiology (STROBE) guidelines.^21^

## Results

### Demographic characteristics

We analyzed a total of 33,439 patients with a diagnosis of HSCT and the prevalence rate of AP was 1.1% (*N* = 359) (Table 1). AP group was comparatively older (5-17 years vs.<5 years) and had more proportion of patients with non-Hispanic Black race and Hispanic ethnicity. The proportion of female patients was higher in the AP cohort when compared to the control population. There was no difference in insurance type between the groups. Also, the AP group was associated with a higher health burden as evidenced by the increased number of chronic conditions ( ≥ 4 vs <4).

**Table 1:** Baseline demographics and clinical characteristics between the study group and the control group

Variable	Class	HSCT with AP	HSCT without AP	*p*
Number of Cases		**359**	**33,080**	
Age (years)	<1 yr	10 (2.8)	2320 (7)	<0.001
	1–4 years	71 (19.8)	10,494 (31.7)	
	5-9 years	104 (29)	8466 (25.6)	
	10-14 years	104 (29)	7164 (21.7)	
	15-17 years	70 (19.5)	4636 (14)	
Gender	Male	184 (51.3)	19,379 (58.6)	0.019
	Female	175 (48.7)	13,694 (41.4)	
Race/Ethnicity	Non-Hispanic White	144 (40.1)	17,349 (52.4)	<0.001
	Non-Hispanic Black	61 (17)	4321 (13.1)	
	Hispanic	93 (25.9)	6280 (19)	
	Asian	18 (5)	1390 (4.2)	
	Other	43 (12)	3740 (11.3)	
Payor	Government	166 (46.2)	14,271 (43.1)	0.496
	Private	138 (38.4)	13,519 (40.9)	
	Other	55 (15.3)	5290 (16)	
Number of CCCs	0-1	231 (64.3)	19587 (59.2)	<0.001
	2–3	71 (19.8)	10,029 (30.3)	
	4+	57 (15.9)	3464 (10.5)	

A *p*-value of <0.05 is noted in bold font.

*AP* acute pancreatitis, *HSCT* hematopoietic stem cell transplantation, *CCC* Complex Chronic Conditions.

We also collected information on underlying etiological conditions and comorbid disorders for both AP and HSCT as reported in the previous national database studies.^11,18^ Of the total 359 patients who had AP, 107 (29.8%) had solid organ transplantation, followed by 85 (23.7%) with primary immunodeficiency disorders (Table 2). Biliary diseases, GVHD, and HLH form the next common disorders with AP. All these disorders were significantly prevalent in the AP group when compared to the HSCT without AP. The complete list of comorbid conditions in univariate analysis in the HSCT population in both groups and their statistical differences in prevalence were noted in Table 2. Among the comorbid conditions, alcohol ingestion, cholangitis, myelodysplastic syndromes, hypercalcemia, inflammatory bowel disease, hemolytic uremic syndrome, abdominal trauma, cystic fibrosis, and diabetic ketoacidosis had a significantly increased proportion of AP.

**Table 2:** Comparison of clinical comorbidities between the groups

Variable	HSCT with AP	HSCT without AP	*p*
Number of patients	**359**	**33,080**	
Solid organ transplant	107 (29.8)	3530 (10.7)	<0.001
Primary immunodeficiency disorders	85 (23.7)	7664 (23.2)	0.820
Graft versus host disease	52 (14.5)	2343 (7.1)	<0.001
Other biliary disease	52 (14.5)	1168 (3.5)	<0.001
Lymphoid leukemia	39 (10.9)	2499 (7.6)	0.019
Choledocholithiasis	28 (7.8)	261 (0.8)	<0.001
Hemophagocytic lymphohistiocytosis	27 (7.5)	844 (2.6)	<0.001
Cholangitis	23 (6.4)	196 (0.6)	<0.001
Myeloid leukemia	21 (5.8)	1976 (6)	0.922
Hypertriglyceridemia	20 (5.6)	583 (1.8)	<0.001
Myelodysplastic syndrome	18 (5)	737 (2.2)	<0.001
Alcohol	16 (4.5)	346 (1)	0.000
Other solid tumors	16 (4.5)	4872 (14.7)	<0.001
Sickle cell disease	11 (3.1)	1012 (3.1)	0.996
Inborn errors of metabolism	8 (2.2)	1176 (3.6)	0.176
Hypercalcemia	8 (2.2)	239 (0.7)	0.001
Inflammatory bowel disease	8 (2.2)	168 (0.5)	<0.001
Thalassemia	3 (0.8)	316 (1)	0.817
Hemolytic uremic syndrome	2 (0.6)	42 (0.1)	0.025
Abdominal trauma	2 (0.6)	46 (0.1)	0.037
Non-Hodgkin’s lymphoma	1 (0.3)	220 (0.7)	0.369
Diabetic ketoacidosis	1 (0.3)	7 (0)	0.002
Cystic fibrosis	1 (0.3)	15 (0)	0.044
Hodgkin’s lymphoma	0(0)	341 (1)	0.053
Aplastic anemias and bone marrow failure syndromes	0(0)	8 (0)	0.768
Systemic lupus erythematosus	0(0)	28 (0.1)	0.581
Anomalies of pancreas	0(0)	5 (0)	0.816

A *p*-value of <0.05 is noted in bold font.

*AP* acute pancreatitis, *HSCT* hematopoietic stem cell transplantation.

### In-hospital mortality and hospital resource utilization

In the entire study population, in-hospital mortality was noted in 1520 patients (4.5%). In unadjusted analysis, the prevalence of in-hospital mortality was much higher in the AP cohort compared to the patients without a diagnosis of AP, 24% (*N* = 86) vs 4.3% (*N* = 1434), *p* < 0.001 (Table 3). In adjusted analysis, AP was confirmed at a higher risk of in-hospital mortality compared to the control group (HR = 1.6; 95% CI: 1.2 to 2.1, *P* < 0.001). The length of stay was higher in the AP group with a median period of 132 days, IQR 55–287 vs 63 days, IQR 22–165, *p* < 0.001. Similarly, the cost of hospitalization was higher in the AP group, 671,821 USD (IQR 23,644–2,004,456) vs 226,190 (IQR 10,395–874,828), *p* < 0.001.

**Table 3:** Unadjusted comparison of clinical outcomes between the two groups

Variable	HSCT with AP	HSCT without AP	*p*
Number of patients	359	33,080	
Length of stay (in days)	132 (55, 287)	63 (22, 165)	<0.001
Hospitalization cost (in USD)	671,821.3 (23,644.3, 2,004,456.4)	226,190 (10,395.1, 847,828.5)	<0.001
Mortality	86 (24)	1434 (4.3)	<0.001
Respiratory failure	139 (38.7)	4003 (12.1)	<0.001
Intubation and mechanical ventilation	125 (34.8)	3118 (9.4)	<0.001
Hypotension	13 (3.6)	903 (2.7)	0.303
Use for vasopressor	2 (0.6)	121 (0.4)	0.551
Acute kidney injury	52 (14.5)	2384 (7.2)	<0.001
Multi-organ dysfunction syndrome	3 (0.8)	30 (0.1)	<0.001
Sepsis	125 (34.8)	3294 (10)	<0.001
Pulmonary embolism	5 (1.4)	63 (0.2)	<0.001
Deep vein thrombosis	13 (3.6)	235 (0.7)	<0.001
Central venous catheter placement	121 (33.7)	6307 (19.1)	<0.001
Parenteral nutrition	238 (66.3)	14,413 (43.6)	<0.001
Pseudocyst	8 (2.2)	13 (0)	<0.001
Percutaneous biliary procedures	1 (0.3)	6 (0)	0.001
Common bile duct exploration	4 (1.1)	14 (0)	<0.001
Cholecystectomy	9 (2.5)	38 (0.1)	<0.001
Endoscopic retrograde cholangiopancreatography	6 (1.7)	31 (0.1)	<0.001
Pancreatectomy	0(0)	5 (0)	0.816

**Fig. 1:**
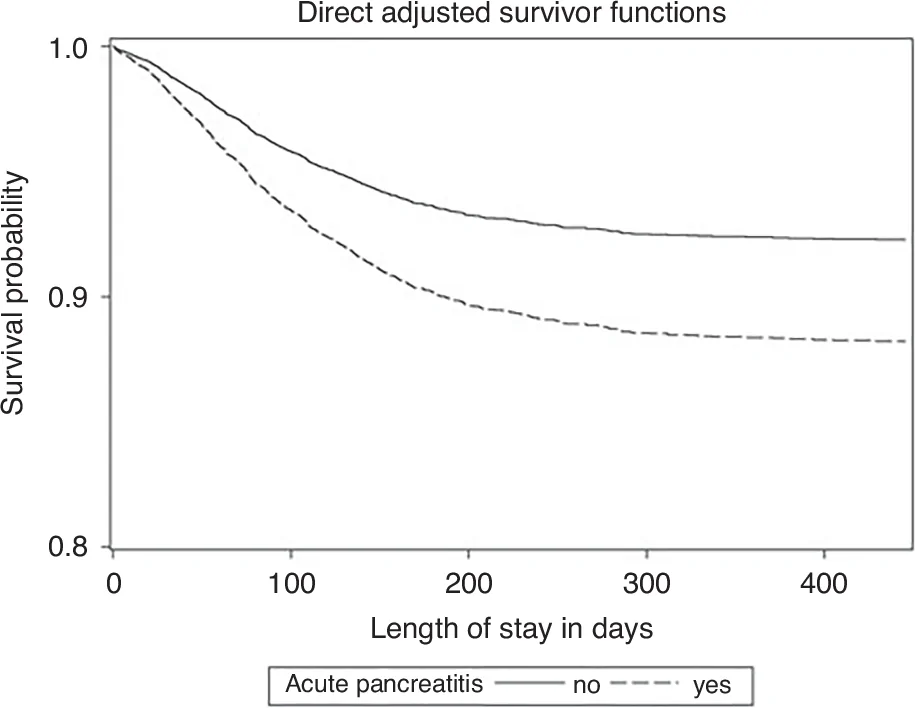
Survival probability. Adjusted Kaplan-Meier curve showing survival probabilities for patients with acute pancreatitis vs. without acute pancreatitis.

The other secondary outcomes evaluated were complications such as respiratory failure, use of intubation and mechanical ventilation, hypotension, use of vasopressors, sepsis, multi-organ dysfunction syndrome, acute kidney injury, pulmonary embolism, and deep vein thrombosis. We also evaluated central venous catheter placement, use of parenteral nutrition, complications, and surgeries/procedures associated with AP or HSCT. Most of these variables were significantly more prevalent in the AP group only with a few exceptions such as the use vasopressors, pulmonary embolism, and pancreatectomy which were not associated with a significant difference between the groups (Table 3). The adjusted Kaplan-Meier curve demonstrated the survival probabilities for patients with acute pancreatitis vs. without acute pancreatitis in Fig. 1.

### Risk stratification model prediction for increased risk of development of AP

Predictive factors were carefully chosen from Tables 1 and 2 for the development of AP in post-HSCT patients to develop a risk stratification model. Choledocholithiasis had the highest score of 14 followed by HLH and older age (10 to 17 years) with a score of 9. Ages between 5 and 9 years were given 8 points. The entire variables used to prognosticate this risk stratification for AP were noted in Table 4. A patient’s total risk score is calculated by summing the points for all present risk factors. Higher scores indicate greater risk. The cut-off point analysis for the total risk score indicates that patients with a total score of >10 have 2.7 (95% CI: 2.2 to 3.4) times the odds of developing AP, with a resulting area under the curve (AUC) of 0.85 (95% CI: 0.84 to 0.88) (Supplementary Fig. S1). Although the shape indicates few false positives, the AUC of 0.85 (95% CI: 0.84 to 0.88) indicates a good and reliable performance. There might be few outliers in some of the features plus cluster overlap that falsely translates in high probability for some cases.

**Table 4:** Multivariable analysis of AP and risk scoring^a^

Variables	Regression Coefficient	OR (95% CI)	*p*	Points
Age Category				
1 to 4 years	0.7767	2.17(1.02,4.64)	0.006	5
5 to 9 years	1.3739	3.95(1.71,9.12)	0.045	8
10 to 14 years	1.4477	4.25(1.85,9.79)	0.001	9
15 to 17 years	1.5063	4.51(1.88,10.84)	0.001	9
Non-White race	0.4157	1.52(1.17,1.97)	0.001	3
Hypertriglyceridemia	0.9889	2.69(1.33,5.44)	0.002	6
Hemophagocytic lymphohistiocytosis	1.4284	4.17(1.95,8.95)	0.006	9
Choledocholithiasis	2.3345	10.33(2.27,47.04)	0.000	14
Myelodysplastic syndrome	0.7813	3.45(1.4,8.45)	0.003	5
Graft versus host disease	0.6228	2.09(1.11,3.92)	0.022	4

^a^Assignment of points to risk factors was based on a linear transformation of the corresponding regression coefficient. The coefficient of each variable was divided by 0.4157 (the lowest coefficient) and multiplied by a constant (2.5) to increase item difference. The final value was rounded to the nearest integer.

## Discussion

Using a national-level database, we demonstrated that the development of AP in pediatric HSCT recipients was associated with adverse outcomes such as increased in-hospital mortality, prolonged length of stay, and higher hospitalization costs. The risk stratification model predicted a high risk of development of AP for a score of >10, with an AUC of 0.85 (95% CI:0.84 to 0.88).

In the present study, we report an AP prevalence rate of 1.11% among HSCT recipients. In previous single-center studies, the prevalence of AP was between 3.5 and 4.4% in bone marrow transplant recipients.^2,12^ In studies involving autopsies of HSCT recipients, AP was noted in approximately one-quarter of patients.^13,14^ This difference in prevalence could be related to multiple factors such as the underlying conditions which required HSCT, the conditioning regimens utilized, and also due to the differences in the methodology utilized. Also, AP could be misdiagnosed or missed as other conditions such as toxicity from conditioning regimens and GVHD may also present with symptoms similar to AP such as abdominal pain, nausea, and vomiting.^1,2,5^ Prior studies have also evaluated the incidence of AP in HSCT. In a single-center study involving 259 children with HSCT, 5% (*N* = 13) developed AP (cumulative incidence) in a median follow-up of 4.4 years.^1^ In a study using the National Inpatient Sample (NIS) database, AP was noted in 0.61% of HSCT-related hospitalizations in adult patients.^11^ In our prior pediatric study using two databases, Kids Inpatient Database (KID) and NIS between 2003 and 2016, AP was noted in approximately 1% of all HSCT-related hospitalizations with an overall increasing trend during the study period.^18^ On the contrary, the epidemiology of AP in general pediatric population is much lower. For, e.g, in a recent global burden of disease study, the age-standardized prevalence rate of pancreatitis in pediatric population was 3.95 (2.75–5.53)/100,000 (95% uncertainty interval).^22^ Similarly, in a nationwide analysis, the incidence of pediatric AP, regardless of etiology was 12.3/100,000 people in 2014 and incidence was stable between the years 2007 and 2014.^23^

AP group was comparatively older (5–17 years vs.<5 years) and had more proportion of patients with non-Hispanic Black race and Hispanic ethnicity which is very similar to prior studies.^11,18^ Both in this study and in our prior study, we noted that increased proportion of female patients in the HSCT recipients with AP whereas in other studies, no difference in sex/gender was noted.^1,11,18^

We demonstrated solid organ transplantation, primary immunodeficiency disorders, biliary diseases, GVHD, HLH, alcohol use, myelodysplastic syndrome, and hyperglyceridemia are the most prevalent conditions associated with HSCT recipients with AP. Among the predictive factors for the development of AP in HSCT recipients in the risk stratification model, we noted that choledocholithiasis had the highest score followed by HLH, age stratification, hypertriglyceridemia, myelodysplastic syndrome, GVHD, and non-White race. Prior studies have noted similar increased associations with these factors.^1,2,4,7,11,18,24–29^ Wang et al noted that pre-existing cholelithiasis, history of donor lymphocyte infusion, younger age ( ≤ 30 years), and GVHD (grades 2–4) as independent risk factors for predicting the development of post-HSCT AP (2). Sajiki et al noted GVHD (either acute or chronic GVHD) in all 13 patients with HSCT who developed AP prior to the onset of AP.^1^ They further noted that acute GVHD (grade 2-4) was an independent risk factor for AP after HSCT (hazard ratio 15.2 (95% CI 4.1–55.8, *p* < 0.001).^1^ Similarly, other studies also noted the association between GVHD and HSCT.^2,7,13,30^

Wang et al reported that patients with AP (moderate to severe AP) were associated with worse outcomes such as reduced overall survival and increased nonrelapse mortality.^4^ In the current study, the prevalence of mortality was higher in the HSCT with AP group compared to patients who did not develop AP (22.7% vs. 4.6%), *p* < 0.001. Wang et al noted a mortality of 7.5% (3/40) attributable to AP and similarly, Ko et al noted 10% mortality due to AP in their cohorts.^4,13^ In multivariate analysis, AP was associated with 1.6 times (95% CI: 1.2 to 2.1, *p* < 0.001) increased risk of mortality. Prior population-based, national-level, epidemiological studies in children and adults have noted that AP-related hospitalizations were independently associated with approximately three times the higher risk of in-hospital mortality in HSCT.^11,18^ In the general population, mortality of AP is much lower compared to HSCT recipients. Pant and colleagues noted that in-hospital mortality of AP was 13.1 per 1000 cases in the year 2000 which decreased to 7.6 per 1000 cases in the year 2009, *P* < 0.001. Similarly, a nationwide study from Japan noted an overall mortality of 0.7% in the pediatric population with AP.^31^ Further, a meta-analysis of 48 studies showed noted that the fatality rate of pediatric AP varied between 3.1–7.4%, which was much lower than the mortality reported in the HSCT recipients.^32^

We noted that the AP cohort overall had increased hospital length of stay, higher cost of hospitalization, increased prevalence of end-organ failures, and other systemic complications in both children and adults with HSCT.^11,18^

We established a risk score model in predicting AP to help identify high-risk patients which will facilitate an early and timely diagnosis of AP in pediatric HSCT recipients. To our knowledge, this is the first study with risk model prediction in pediatric patients. We used age, race, and coexisting/underlying conditions (Table 4). Similarly, Wang et al reported a risk score in adult HSCT recipients using GVHD (grades 2–4), younger age adults, and a history of donor lymphocyte infusion to be independent risk factors for predicting the development of post-HSCT AP (2). Other conditions which can predispose to AP in post-HSCT such as infections, GVHD, hyperglyceridemia, alcohol, and medications used in conditioning or for immunosuppression. Clinicians should be vigilant in screening for these conditions prior to- and post-HSCT and manage them aggressively to prevent AP. For example, elective cholecystectomies can be considered prior to HSCT in patients with preexisting gallstones. Prevention or early aggressive management of AP can lead to decreased healthcare utilization and improvement in mortality.

We acknowledge the several limitations of this study. Given the retrospective nature, we did not have access to pertinent detailed information such as laboratory values (pancreatic enzyme levels, etiological tests for AP, etc..) imaging findings, severity of AP, specific management details of AP, and other details of HSCT (donor details, HLA matching, conditioning regimen, initial HSCT vs subsequent HSCT, time from HSCT to AP, details of GVHD prophylaxis etc..). Given the methodology utilized here, we cannot infer causality, and the predictive risk scoring is derived based on the strong association. As we rely on accurate coding and accurate documentation, coding errors might have affected our results and interpretation. Due to the lack of specific ICD codes, we cannot differentiate if the pancreatitis episodes were primary or recurrent. Any medical care received outside of the hospital admissions in the CHA was also unavailable in the database, further limiting the assessment of the AP disease course.

Despite these limitations, our study has several strengths as well. The inclusion of a large sample from 49 hospitals at the national level minimized the sample bias from single-center studies and a major strength of this study. We also utilized multivariable regression to minimize confounding factors. We also employed multiple, frequent quality checks at different points of data collection to ensure the reliability of the data through the PHIS database. We also predicted a novel risk stratification model, which predicted 2.7 (95% CI: 2.2 to 3.4) increased risk of AP for a score of >10, with an AUC of 0.85 (95% CI: 0.84 to 0.88).

## Conclusion

AP in the HSCT population prognosticates adverse outcomes with increased in-hospital mortality, prolonged hospital stay, and higher hospitalization costs. Clinicians managing patients with HSCT need to be vigilant in both anticipation and aggressive management of AP. Specific gastroenterology and hemato-oncology comorbidities significantly increase the risk of AP, thus warranting clinicians to anticipate and take appropriate preventive strategies for all modifiable and preventable risk factors for AP. Our findings should be validated in a prospective multicenter cohort that captures more comprehensive clinical data.

## Supplementary information

Supplemental figure

Supplemental table

## Data Availability

All data generated or analyzed during this study are included in this published article and the supplementary information files.
