# Supplementary material for: Acute pancreatitis adversely impacts the outcome in hospitalized pediatric hematopoietic stem cell transplantation recipients
Source: Pediatr Res. 2026 Jan 16;100(3):1365–71. doi: 10.1038/s41390-025-04753-z (PMC13672041; doi:10.1038/s41390-025-04753-z)

Figure 2 – Receiver operating characteristic curve (ROC) for prediction of acute pancreatitis based on variables listed in Table 4.

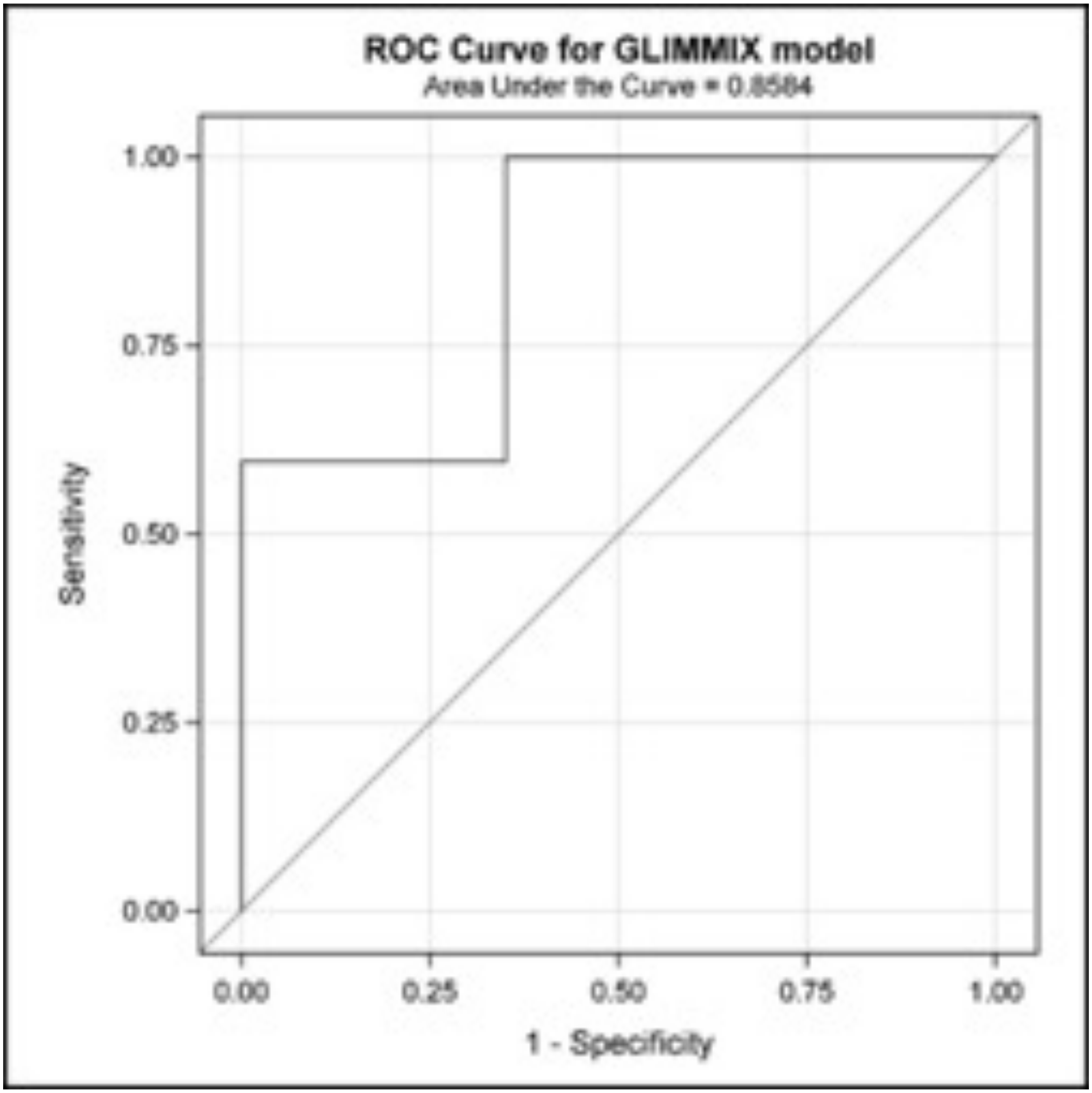

Supplement: Supplementary file 1 [file 41390_2025_4753_MOESM1_ESM.pdf]
