# Supplementary material for: Acute pancreatitis adversely impacts the outcome in hospitalized pediatric hematopoietic stem cell transplantation recipients
Source: Pediatr Res. 2026 Jan 16;100(3):1365–71. doi: 10.1038/s41390-025-04753-z (PMC13672041; doi:10.1038/s41390-025-04753-z)
Supplement: Supplementary file 2 [file 41390_2025_4753_MOESM2_ESM.pdf]

***International Classification of Diseases, 9th Revision and 10th Revision, Clinical Modification* (ICD-9/10-CM), and Current Procedural Terminology codes**

Inclusion Criteria - Hematopoietic stem cell transplantation-related diagnostic and procedural codes

		ICD 9	ICD 10
Any one of the diagnosis or procedure codes	Diagnosis Codes	V4281; V4282; 99685; 99688	Z9481;Z9484;T8600;T8601;T8602;T8603;T8609;Z48290;T865
	Procedure codes	4100;4102; 4103;4104; 4105;4106; 4107;4108; 4109	30243AZ;30243G0;30240G1;30243G2;30243G3; 30243G4;30243X0;30240X1;30243X2;30243X3; 30243X4;30243Y0;30240Y1;30243Y2;30243Y3;30243Y4; 30243U0;30243U1;30243U2;30243U3;30243U4; 30240AZ;30240G0;30240G1;30240G2;30240G3; 30240G4;30240X0;30240X1;30240X2;30240X3; 30240X4;30240Y0;30240Y1;30240Y2;30240Y3; 30240Y4;30240U0;30240U1;30240U2;30240U3; 30240U4; 30233AZ;30233G0;30240G1;30233G2;30233G3; 30233G4; 30233X0;30240X1;30233X2;30233X3;30233X4;30233Y0; 30240Y1;30233Y2;30233Y3;30233Y4;30233U0;30233U1; 30233U2;30233U3;30233U4; 30230AZ;30230G0; 30240G1;30230G2;30230G3;30230G4; 30230X0;30240X1;30230X2;30230X3;30230X4;30230Y0; 30240Y1;30230Y2;30230Y3;30230Y4;30230U0;30230U1; 30230U2;30230U3;30230U4

Exclusion criteria

Any patient with chronic pancreatitis

ICD 9	ICD 10
5771	K860;K861

Primary exposure

Acute pancreatitis at any encounter during or following BMT hospitalization

ICD 9	ICD 10
5770	K85%

Covariates related to BMT

	ICD 9 codes	ICD 10 Codes
Myelodysplastic syndrome	23874;23875;23876	D46%
Lymphoid Leukemia	2.04	C91%
Myeloid Leukemia	2.05	C92%
Hodgkin's Lymphoma	2.01	C81%
Non-Hodgkin's lymphoma	2020%;2027%;2028%;2029%	C85%
Other solid Tumors	1940;1905;170%;1890;1830;191%;192%	C69%;C74%;C40%;C64%;C62%;C56%;C70%;C71%;C72%
Aplastic anemias and Bone marrow failure syndromes	2.84	D60%

HLH	2884	D761;D762;D763
Thalassemia	28.24	D56%
Sickle Cell Disease	28.26	D57%
Inborn Errors of Metabolism	2775;2727;3300;27786	E76%;E75%;E7152%
Primary Immunodeficiency disorders	2790 to 2793	D80% to D84%
GVHD	27.95	D8981%

Covariates Related to Acute Pancreatitis

Hemolytic Uremic Syndrome	28311	D593
Cholangitis	576.1	K83.*
Choledocholithiasis	574*	K80.*
Other Biliary disease	576.2, 576.8, 782.4, 576.9	K83.1, K83.8, K83.9, R17
Hypertriglyceridemia	272.1	E78.1
Hypercalcemia	275.42	E83.52
Inflammatory bowel disease	555*, 556*	K50*, K51*

Systemic Lupus Erythematosus	695.4, 710.0	M32*, L93*
Diabetic Ketoacidosis	250.1*	E081, E0811, E091, E0911, E101, E1011, E111, E1111
Cystic Fibrosis	277.0*	E84*
Alcohol	291*, 303*, 305*, 760.71, 980.0, 357.5, 425.5, 535.30, 535.31, 571*	G62.1, G31.2, T510X1A, T510X2A, T510X3A, T510X4A, F10*, Y90*, K70*, Z714*, K29.0, K29.1, K29.2.
Abdominal Trauma	863*, 864*, 865*, 866*, 867*, 868*, 869*.	S36 * to S39*
Solid organ transplant	996.8*, V42 to V42.7, V42.84, , V 42.83, Z94.83, V42.9	T86.1*, T86.2*, T86.3*, T86.4*
Anomalies of pancreas	751.7	Q45.0 to Q45.3
Cystic Fibrosis	277.0*	E84*

Other Complications - This may go with the outcomes table 2

Acute Kidney Injury	5.84	N17%
Respiratory failure	518.0, 518.81, 518.82, 518.84	J96.0*, J96.2*, J98.1*
Intubation and Mechanical Ventilation (procedure codes)	93.90;96.0%;96.7%	5A09357, 5A09457, 5A09557, 09HN7BZ, 0CHY7BZ, 0DH57BZ, 0NH17EZ, 5A1935Z, 5A1945Z, 5A1955Z, 0BH07DZ

Hypotension	4588;4589	I950;I959;I9589
Use for vasopressor (procedure codes)	"0017"	3E030XZ;3E030XZ; 3E043XZ;3E040XZ; 3E050XZ;3E053XZ; 3E063XZ;3E060XZ
Multi-Organ dysfunction Syndrome	99594	R6511
Sepsis	77181;99591;99592	A021;A227;A267;A327;A400;A401;A403;A408; A409;A4101;A4102;A411;A412;A413;A414;A4150; A4151;A4152;A4153;A4159;A4181;A4189;A419; A427;A5486;B377;P360;P3610;P3619;P362; P3630;P3639;P364;P365;P368;P369;R6520; R6521;T8144XA;T8144XD;T8144XS
PE	415.1*	I26*
DVT	453.4*, 453.82, 453.82	I824*, I826*
Central Venous Catheter placement (procedure codes)	38.93, 86.07	0JH63XZ, 0JHD3XZ, 0JHF3WZ, 0JHM3XZ, 0JHL3XZ
Parenteral Nutrition		
**Pancreatitis related complications and procedures**		
Pseudocyst	5772	K86.3

Percutaneous biliary procedures (procedure codes)	51.01, 51.96, 51.98	0F943ZZ, 0FC93ZZ, 0F753Dz, 0F753DZ, 0F63ZZ, 0F783DZ, 0F783ZZ, 0F793ZZ, 0F793DZ, 0F993ZZ, 0F994ZZ, 0FC53ZZ, 0FC54ZZ, 0FC64ZZ, 0FC84ZZ, 0FC84ZZ, 0FC83ZZ, 0FC63ZZ.
Common bile duct exploration (procedure codes)	51.02, 51.03, 51.04, 41.32, 51.36, 51.37, 51.39, 51.41, 51.43, 51.51, 51.59, 51.63, 51.64, 51.69, 51.71, 51.79	0FT90ZZ, 0FT50ZZ, 0FT60ZZ, 0FT70ZZ, 0FT80ZZ, 0F550ZZ, 0F560ZZ, 0F570ZZ, 0F580ZZ, 0FB50ZZ, 0FB60ZZ, 0FB70ZZ, 0FB80ZZ, 0FC90ZZ, 0F9900Z, 0F9900Z, 0F990ZZ, 0F9940Z, OF9400Z, 0FC400Z, 0FQ97ZZ, 0FQ90ZZ, 0F150D3, 0F150DB, 0F150Z3, 0F150ZB, 0F160D3, 0F160DB, 0F160Z3, 0F160ZB, 0F170D3, 0F170DB, 0F170Z3, 0F170ZB, 0F180D3, 0F180DB, 0F180Z3, 0F180ZB, 0F190D3, 0F190DB, 0F190Z3, 0F190ZB, 0F140D3, 0F140DB, 0F140Z3, 0F140ZB,
Cholecystectomy (procedure codes)	51.2*	0FB40ZX, 0FB40ZZ, 0FB43ZX, 0FB43ZZ, 0FB48ZX, 0FB48ZZ, 0FB44ZX, 0FB44ZZ,
ERCP (procedure codes)	51.83, 51.84, 51.85, 51.86, 51.87, 51.88, 51.10, 51.11, 51.14, 52.13, 52.93, 52.94, 52.98, 97.05.	0FQC7ZZ, 0FQC8ZZ, 0F758ZZ, 0F768ZZ, 0F778ZZ, 0F7C8DZ, 0F7C8ZZ, 0F958ZZ, 0F968ZZ, 0F978ZZ, 0F988ZZ, 0F998ZZ, 0F9C8ZZ, 0F9D8ZZ, 0F9C80Z, 0F9980Z, 0F758DZ, 0F768DZ, 0F778DZ,

		0F788DZ, 0F798DZ, 0FHB4DZ, 0FHB8DZ, 0FC58ZZ, 0FC68ZZ, 0FC78ZZ, 0FC98ZZ, 0FC88ZZ, 0FCC8ZZ, 0FF48ZZ, 0FF58ZZ, 0FF68ZZ, 0FF78ZZ, 0FF88ZZ, 0FF98ZZ, 0FFC8ZZ, 0F959ZX, 0F968ZX, 0F978ZX, 0F988ZX, 0F998ZX, 0F9C8ZX, 0FB48ZX, 0FB58ZX, 0FB68ZX, 0FB78ZX, 0FB88ZX, 0FB98ZX, 0FBC8ZX, 0FHD8DZ, 0FCD8ZZ, 0F7D8ZZ, 0F758DZ, 0F778DZ, 0F798DZ, 0F788DZ, 0F7D8DZ, 0FHB8DZ, 0FPB8DZ,0FPD8DZ
Pancreatecto my (procedure codes)	52.22, 52.95, 52.96 52.99	0F7D0ZZ, 0FQD0ZZ, 0FQG0ZZ, 0F1D0D3, 0F1D0DB, 0F1D0Z3, 0F1D0ZB, 0F1G0D3, 0F1G0ZB, 0F1G0ZZ, OFTG0ZZ, 0F1G0ZC, 0FTG0ZZ, 0FBG0ZZ, 0F9G00Z, 0FCD0ZZ, 0FCD8ZZ, 0F9D0ZX, 0F9D8ZZ
